# SEXUAL AND GENDER IDENTITY AND THEIR PROPERTIES IN AGING WITH PRIDE: NATIONAL HEALTH, AGING, AND SEXUALITY AND GENDER STUDY

**DOI:** 10.1093/geroni/igad104.0705

**Published:** 2023-12-21

**Authors:** Christi Nelson, Karen Fredriksen-Goldsen, Charles Hoy-Ellis, Meghan Romanelli, Hyun-Jun Kim

**Affiliations:** University of Washington, Seattle, Washington, United States; University of Washington, Seattle, Washington, United States; University of Washington, Seattle, Washington, United States; University of Washington, Seattle, Washington, United States; University of Washington, Seattle, Washington, United States

## Abstract

Increasing diversity and rapidly evolving sociopolitical context are changing how sexuality and gender identity are experienced, expressed, and measured in contemporary America. Based on longitudinal data from Aging with Pride: National Health, Aging, and Sexuality/Gender Study, we examine sexual and gender identity, differentiation from other related constructs (i.e., desire, behavior, expression, relationships) as well as the associations between properties of sexual and gender identities (e.g., congruence, continua, visibility, centrality, and transformation) and the health and well-being of LGBTQ midlife and older adults (N=2,233), aged 50-102 at baseline. The findings document important differences in current as well as lifetime sexual experiences, desires, and romantic relationships by sexuality and gender identity. Measures incorporating a continuum as opposed to binary response categories better capture the experiences of bisexual, sexually diverse, and transgender older participants as compared to lesbians and gay men. When examining transformation, 7% reported changes to their sexual identity and 3% reported changes to their gender identity over 4 years. Those who changed their identity over time showed a higher level of identity stigma and poorer physical and psychological health-related quality of life. Our findings reveal that heterogeneity and intersectionality are critical to understanding sexual and gender identity. It is important to consider multiple aspects of sexuality and gender (e.g., romantic relationships) that are often overlooked in gerontological and sexuality and gender research. Scholarship on sexual and gender identity development and transformation in later life is needed to better address diversity in the aging population.

